# Psychoanalysis, Philosophy and Literature- Intersection of Science and Art

**DOI:** 10.1192/j.eurpsy.2023.2069

**Published:** 2023-07-19

**Authors:** D. Kolar, M. Kolar

**Affiliations:** 1 Psychiatry, Queen’s University, Kingston, Ontario, Canada; 2 Queen’s University, Kingston, Canada

## Abstract

**Introduction:**

Philosophy and psychoanalysis have mutually influenced each other in many ways. Ancient Greek philosophers, Socrates and Plato were frequently cited by Freud in his works and the origins of certain psychoanalytic concepts can be found in their works. The philosophical works of Kant, Schopenhauer, Nietzsche, Heidegger, Husserl, Sartre and many others had a significant impact on the development of psychoanalytic ideas. The intersection of philosophy and literature was best depicted in Simone de Beauvoir’s concept of the metaphysical novel.

**Objectives:**

The goal of this presentation is to perform a comprehensive historical review of the relationship between psychoanalysis, philosophy and literature.

**Methods:**

Different philosophical schools from ancient philosophy to classic German philosophy and philosophy of existentialism have been explored in their relationship with psychoanalysis and world literature. Among world literary classics, we selected only those who best represent the role of psychoanalysis in the modern literary critics and on the other hand the influence of philosophy on literature.

**Results:**

Early origins of the relationship between philosophy, psychoanalysis and literature can be found in the text of ancient philosophers and writers. The great Sophocles’ tragic drama Oedipus the King was the foundation for Freud’s concept of Oedipus complex. The Socratic dialogue, a technique best elaborated by his student Plato was the antecedent of modern psychotherapy. Later in history philosophical works of Kant, Schopenhauer, Nietzsche, Husserl, Heidegger, Sartre and many others had a significant impact on the development of psychoanalytic ideas. There is a number of other philosophical fictions in the world literature written by Sartre, Camus, Kafka, Proust and many others and some of these literary woks may have characteristics of psychological novel as well. Literary critics is an important field for the application of psychoanalysis. Psychoanalytic theory has been always in forefront of Shakespearean studies. Marcel Proust is a writer who gave a significant contribution to modern literary studies. He wrote about the interactive process between the reader and text and emotional impact of reading. Proust recognized the similar psychological processes that we can see in psychoanalytic setting.

**Conclusions:**

This comprehensive historical review of the relationship between psychoanalysis, philosophy and literature demonstrates that all these disciplines have much in common, particularly in their intention to approach truth from different angles. Psychoanalysis is a science and applies scientific methodology in its theory and treatment. Certain branches of psychoanalysis like Jung’s analytic psychology are sometimes closer to philosophy and art than to science. Philosophy as a humanistic discipline has always been in between science and art.

**Disclosure of Interest:**

None Declared

